# An Examination of the Impact of Clergy-Involved Mental Health Activities for Their Congregants on Clergy Life Satisfaction, Happiness, and Perceptions of Having a Life Close to Ideal in the USA

**DOI:** 10.1177/15423050241268397

**Published:** 2024-08-02

**Authors:** Augustine Cassis Obeng Boateng, Katherine Carroll Britt, Joshua Sebu, Hayoung Oh

**Affiliations:** Department of Biobehavioral Sciences, 16142 University of Pennsylvania School of Nursing, Philadelphia, PA, USA; Spirituality & Health Hub, Philadelphia, PA, USA; Department of Biobehavioral Sciences, 16142 University of Pennsylvania School of Nursing, Philadelphia, PA, USA; Spirituality & Health Hub, Philadelphia, PA, USA; Spirituality & Health Hub, Philadelphia, PA, USA; Department of Data Science and Economic Policy, 107841 University of Cape Coast, Cape Coast, Ghana; Spirituality & Health Hub, Philadelphia, PA, USA

**Keywords:** Clergy, congregants, happiness, life satisfaction, mental health needs

## Abstract

Clergy play a crucial role in supporting the mental health of their congregants, but the impact on their own well-being is understudied. A review of 2019–2020 data from 636 U.S. religious leaders using generalized estimating equations analysis found that clergy use prayer, preaching, readings on mental health, and referrals to mental health professionals to support congregants’ well-being. Future longitudinal studies are needed to understand the needs of diverse clergy groups.

## Introduction

As the clergy's role in supporting mental health in America continues to evolve (Boateng et al., 2024), there is a growing emphasis on various aspects of their work, including providing spiritual and emotional support, promoting mental health awareness, offering counseling services to community members, and facilitating access to necessary mental health resources (Ellison et al., 2006; Hays, 2015; Heseltine-Carp & Hoskins, 2020; Hirono & Blake, 2017; Iheanacho et al., 2021). However, there is a limited understanding of how these increasing demands might affect clergy members’ happiness and life satisfaction, and the specific activities they engage in to navigate the challenges (Terry & Cunningham, 2020) for which they may be untrained.

Clergy members face significant mental health demands from their congregants (Hartford University, 2024), which can impact their happiness and life satisfaction. On the one hand, clergy can find great satisfaction in helping people with mental health challenges (Hays & Shepard Payne, 2020). Seeing someone heal and grow can give clergy a sense of purpose and satisfaction. Eventually, this can translate into increased happiness and job satisfaction, knowing they are fulfilling their calling and making a difference in their congregants’ lives (Miner et al., 2010). A survey of United Methodist clergy members in the USA found that high levels of spirituality were associated with greater personal accomplishment. However, high levels of spirituality also increased emotional exhaustion and depersonalization (Doolittle, 2007).

The mental health demands of clergy work can be emotionally, psychologically, and spiritually taxing (Hileman, 2008). Evidence suggests that clergy members, because they are constantly exposed to the struggles and suffering of their congregants, are at an increased risk of mental health problems themselves, including depression, anxiety (Proeschold-Bell et al., 2013), burnout (Clarke et al., 2023), and substance abuse (Frenk et al., 2013), and are more likely to experience vicarious trauma, compassion fatigue (Hendron et al., 2014), and emotional exhaustion (Hileman, 2008). They may feel overwhelmed by the weight of others’ pain and find it difficult to set personal boundaries or engage in self-care. Additionally, dealing with complex mental health issues may require specialized knowledge and skills that clergy members may not possess, leading to feelings of inadequacy or frustration (Edwards et al., 2021). Specifically, previous research (Stephens, 2020) shows that clergy with less mental health training may feel pressured to provide help that they are not qualified to give, or they may feel overwhelmed by the number of people seeking their support which may compound existing feelings of stress and even depression (Case et al., 2020).

It is important to highlight that the observed correlation between the demands of clergy roles and adverse mental health outcomes mirrors what has been documented in the broader population. For instance, a study by Holleman and Eagle (2023) revealed that mental health outcomes among clergy were similar to those in the general population. The authors posit that the notable negative mental health outcomes observed among clergy may be attributed to methodological limitations in prior studies and the predominant overrepresentation of individuals from mainline Protestant denominations (Holleman & Eagle, 2023). Regardless, given the distinct role clergy play in supporting their congregants, even minor mental health challenges may influence their ability to fully support different aspects of their ministry.

Besides the inherent challenges of supporting congregants with mental health concerns, clergy members may encounter additional obstacles, such as stigma and cultural barriers within their religious communities (Jacobi et al., 2022; Wesselmann & Graziano, 2010). These barriers can create a challenging environment for clergy members to address mental health issues or advocate for professional assistance openly. Such a challenging environment can give rise to tension and internal conflict (Leavey et al., 2007), ultimately affecting clergy members’ happiness and life satisfaction. Furthermore, if clergy members lack the necessary mental health resources, they may find it challenging to meet the mental health needs of their congregants effectively (Leavey et al., 2007). The lack of mental health resources may compound feelings of inadequacy, stress, and burden, further impacting their emotional state and overall life satisfaction and happiness.

In addition, negative stereotypes and biases held between mental health professionals and clergy can make it difficult for the two groups to collaborate and communicate effectively (Sullivan et al., 2014). This can impede referrals and access to mental health services and limit the options available to congregants seeking help. If clergy are less likely to refer individuals to mental health professionals, this can increase the workload of the clergy and put additional strain on them and place congregants at risk of not getting the mental health attention they need. Investigating the mental health activities that clergy participate in to address the needs of their congregants may open doors to improved interventions and collaboration with secular mental health providers.

This study is informed by the Job Demands-Resources Model, a theory that explains how the demands and resources of a job can affect an individual's well-being (Bakker & Demerouti, 2007). This theoretical framework suggests a clear distinction between job demands and resources, each having unique impacts on well-being. Job demands encompass a job's physical, psychological, and social aspects that necessitate consistent effort and are linked to physiological and psychological strains. On the other hand, job resources refer to a job's physical, psychological, and social aspects that assist individuals in managing job demands and accomplishing their work objectives (Demerouti & Verbeke, 2004). By exploring the interaction between ministerial demands and the resources at their disposal, this framework can shed light on the intricate nature of the challenges that clergy members face and how they address them.

Using 2019–2020 data from the National Survey of Religious Leaders study, this study aims to examine the impact of clergy-involved mental health activities for their congregants on clergy life satisfaction, happiness, and perceptions of having a life close to ideal (*the extent to which a clergy perceives his/her life as being close to their personal ideals or aligns with their values and inspirations*). Understanding how the mental health needs of congregants affect the life satisfaction and happiness of the clergy can create an understanding of the challenges and complexities of their role as well as the support system and resources that allow them to thrive.

**Hypothesis:** There is an association between clergy-involved mental health activities for congregants and clergy life satisfaction, happiness, and life close to ideal.

## Methods

### *Data*

This study utilizes data from the National Survey of Religious Leaders (NSRL) conducted by NORC at the University of Chicago between February 2019 and June 2020 (Chaves et al., 2022). The data collection coincided with the fourth wave of the National Congregations Study (NCS-IV) and involved an online self-administered survey questionnaire. The NSRL data represents a nationally representative sample of religious leaders of religious congregations across various religious traditions. The operational definition of religious leaders in this study includes those engaged in key religious activities such as preaching, teaching, leading worship services, conducting rituals, and providing pastoral care.

**Figure 1.**
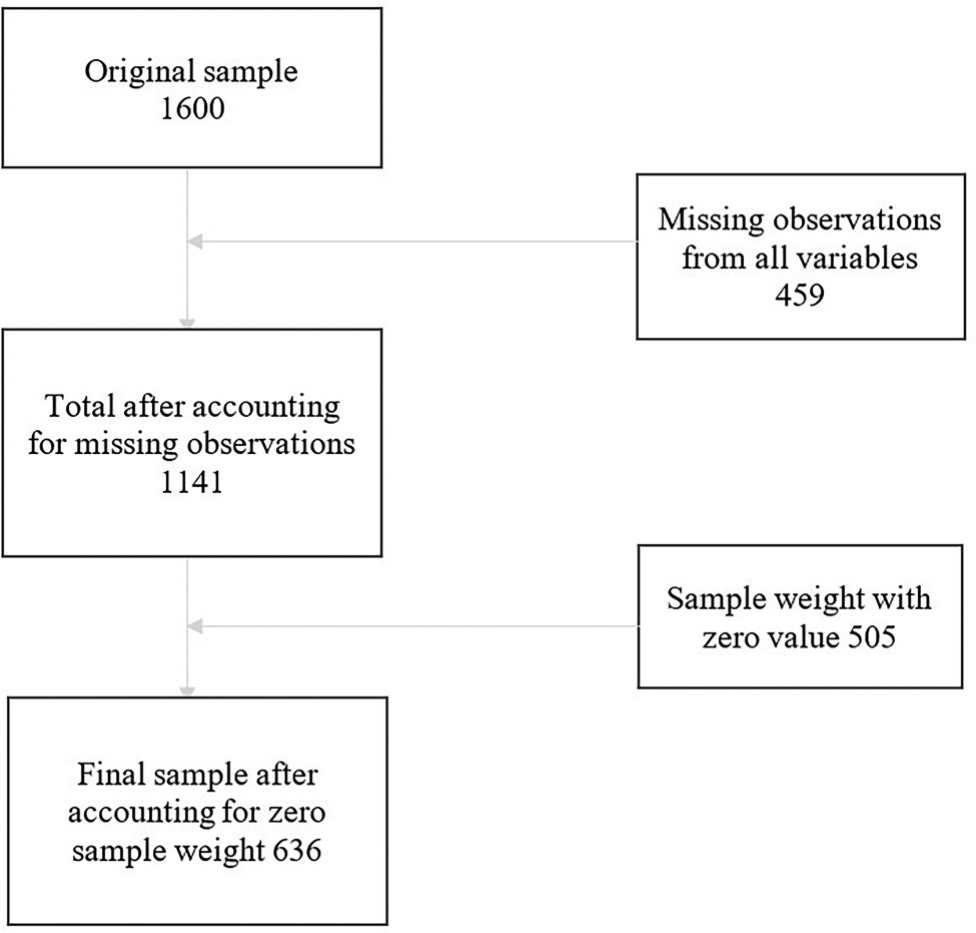
Flow chart.

The sample comprises paid and unpaid primary leaders serving congregations and paid secondary leaders involved in religious work and serving congregations of up to 25 members. The final dataset consists of 1,600 religious leaders, with 890 primary leaders. According to Chaves et al. (2022), the primary-leader sample exhibits higher quality due to better response rates and less nonresponse bias. Therefore, in line with Chaves et al.'s recommendation, this study exclusively utilizes the primary-leader sample and recommended sample weights. Figure 1 explains how the final sample size of 636 used for the estimation was obtained.

### *Measures*

*Subjective well-being:* Outcome variables are the clergy's self-reported measures of happiness (with responses: Every day; Almost every day; Two or three times a week; About once a week; Once or twice; Never), life satisfaction (with responses: Every day; Almost every day; Two or three times a week; About once a week; Once or twice; Never), and life's being close to ideal (with responses: Completely agree; Moderately agree; Slightly agree; Neither agree nor disagree; Slightly disagree; Moderately disagree; Completely disagree).

All outcome variables were dichotomized as follows: Happy (Every day; Almost every day) and Not happy (Two or three times a week, About once a week; Once or twice; Never); Satisfied with life (Every day; Almost every day) and Not satisfied with life (Two or three times a week, About once a week; Once or twice; Never); life close to ideal (Completely agree; Moderately agree); and life not close to ideal (Slightly agree, Neither agree nor disagree; Slightly disagree; Moderately disagree; Completely disagree).

*Mental Health Activities:* Variables of interest included mental health resources either used or practiced. As shown in Table 1, these included whether a religious leader has been asked for help, encouraged someone to seek help from a mental health professional, preached a sermon on mental illness, led a prayer or healing focused on mental illness, organized a class studying mental illness or read a book, attended a class, or looked up resources on mental illness all in the last 12 months. These were dichotomized into Not at all and Once or more.

**Table 1.** Definition of Variables.

Variables	Definition
Happy	Happy = 1 (Every day; Almost every day) and Not happy = 0 (Two or three times a week; About once a week; Once or twice; Never)
Satisfied with life	Satisfied with life = 1 (Every day; Almost every day) and Not satisfied with life = 0 (Two or three times a week; About once a week; Once or twice; Never)
Life close to ideal	Life close to ideal = 1 (Completely agree; Moderately agree) and Life not close to ideal = 0 (Slightly agree; Neither agree nor disagree; Slightly disagree; Moderately disagree; Completely disagree)
Female	Female = 1, Male = 0
BMI	Normal = 1; Underweight/Overweight/Obese = 0
Help with Mental Illness	In past 12 months how often has been asked for help with mental illness (0 = Not at all, 1 = Once or more)
Mental Health referral	How often encouraged someone to seek help from mental health professional in last 12 months (0 = Not at all, 1 = Once or more)
Sermon on Mental Illness	How often preached a sermon on mental illness in last 12 months (0 = Not at all, 1 = Once or more)
Prayer on Mental Illness	How often led prayer or healing focused on mental illness in last 12 months 0 = Not at all, 1 = Once or more
Class on Mental Illness	How often organized a class studying mental illness in last 12 months 0 = Not at all, 1 = Once or more
Education on Mental Illness	How often read a book, attended a class, or looked up resources on mental illness in last 12 months 0 = Not at all, 1 = Once or more
Married	1 = Married, 2 = Not Married
Education	
No College or formal pastoral or ministerial training	1 = Yes; 0 = No
Any formal pastoral or ministerial training	1 = Yes; 0 = No
Bachelor's degree	1 = Yes; 0 = No
Graduate Degree (no mdiv)	1 = Yes; 0 = No
mdiv	1 = Yes; 0 = No
Race	
White or Am Ind	1 = Yes; 0 = No
Black	1 = Yes; 0 = No
Hispanic	1 = Yes; 0 = No
Asian/PI	1 = Yes; 0 = No
COVID	Data gathered after the onset of the COVID-19 pandemic 1 = Yes; 0 = No
Hours spent on religious activity/week	Total hours spent on all work activities in a typical week
Other streams of income	Has income from sources other than congregation. 1 = Yes; 0 = No
Financial satisfaction	Satisfaction with financial situation. 1 = Not at all satisfied; 2 = More or less satisfied; 3 = Pretty well satisfied
Contemplated leaving religious work	How often considered leaving religious work. Never = 0; once in a while to often = 1.
Doubts about faith	How often R has doubts about their religious faith. Never or Don’t have a religious faith = 0; Sometimes to all the time = 1.
Feeling cared for	Extent R feels cared for by congregation. Low (Not at all to moderate) = 0; High (Quite a bit and very much) = 1
Religious Tradition	
White conservative, evangelical, or fundamentalist	1 = Yes; 0 = No
Black Protestant	1 = Yes; 0 = No
White liberal or moderate	1 = Yes; 0 = No
Non-Christian	1 = Yes; 0 = No

*Covariates:* Evidence suggests that several factors including sex (Holleman & Eagle, 2023), education level (Dougherty et al., 2020), race (Schleifer & Cadge, 2019), religious tradition (Holleman & Eagle, 2023), faith, body mass index (BMI) (Holleman & Eagle, 2023; Yao et al., 2023), time of data collection (Losch et al., 2002), income, and social support (Proeschold-Bell et al., 2015) can influence the work of clergy members and by proxy their well-being. As such these variables were controlled in the analysis. For example, the following variables from the original dataset were categorized as follows: sex (male/female), education level (no college or formal pastoral/ministerial training, all degrees except MDiv, formal pastoral training), race (White or American Indian, Black, Hispanic, Asian/Pacific Islander), religious tradition (Black Protestant, White liberal or moderate, Non-Christian), and income (total household income categorized as low, middle, and high). For a detailed explanation of all variables, see Table 1.

*Ethics:* This secondary data analysis is exempt from Institutional Review Board (IRB) review at the University of Pennsylvania because it involves de-identified and uncoded data of participants in the original study.

### *Statistical Analysis*

A binomial generalized linear model (GLM) was employed to analyze the data. The GLM is a statistical framework that investigates the association between the expected value of a response variable and a linear combination of explanatory variables (McCullagh, 2019; Müller, 2012). When dealing with a binomially distributed dependent variable, as in this analysis, the GLM establishes a fixed relationship between the conditional expectation of the probability of a single Bernoulli trial and a specific value. In line with this approach, the study utilizes the logistic (log-odds) link function, which enables the expression of results as the ratio of the probability of success to the probability of failure. Results are presented in the odds ratio for better interpretation.

### *Analytical Plan*

A complete-case analysis technique (Little et al., 2022) was used to address missingness in the dataset. Therefore, the sample exclusively comprises cases with complete and valid data of variables under consideration. After applying the suggested sample weights, 636 cases were included in the analysis. See flowchart in Figure 1. In addition, due to the data being skewed to the left, the variables were dichotomized after a series of checks. For example, only 17.07% of respondents reported “Never” to being “Happy two or three times a week.” Therefore, we made the determination that only individuals who reported being “happy almost every day” or “happy every day” would be considered as “happy,” while the remainder would be classified as “not happy.”

## Results

**Figure 2.**
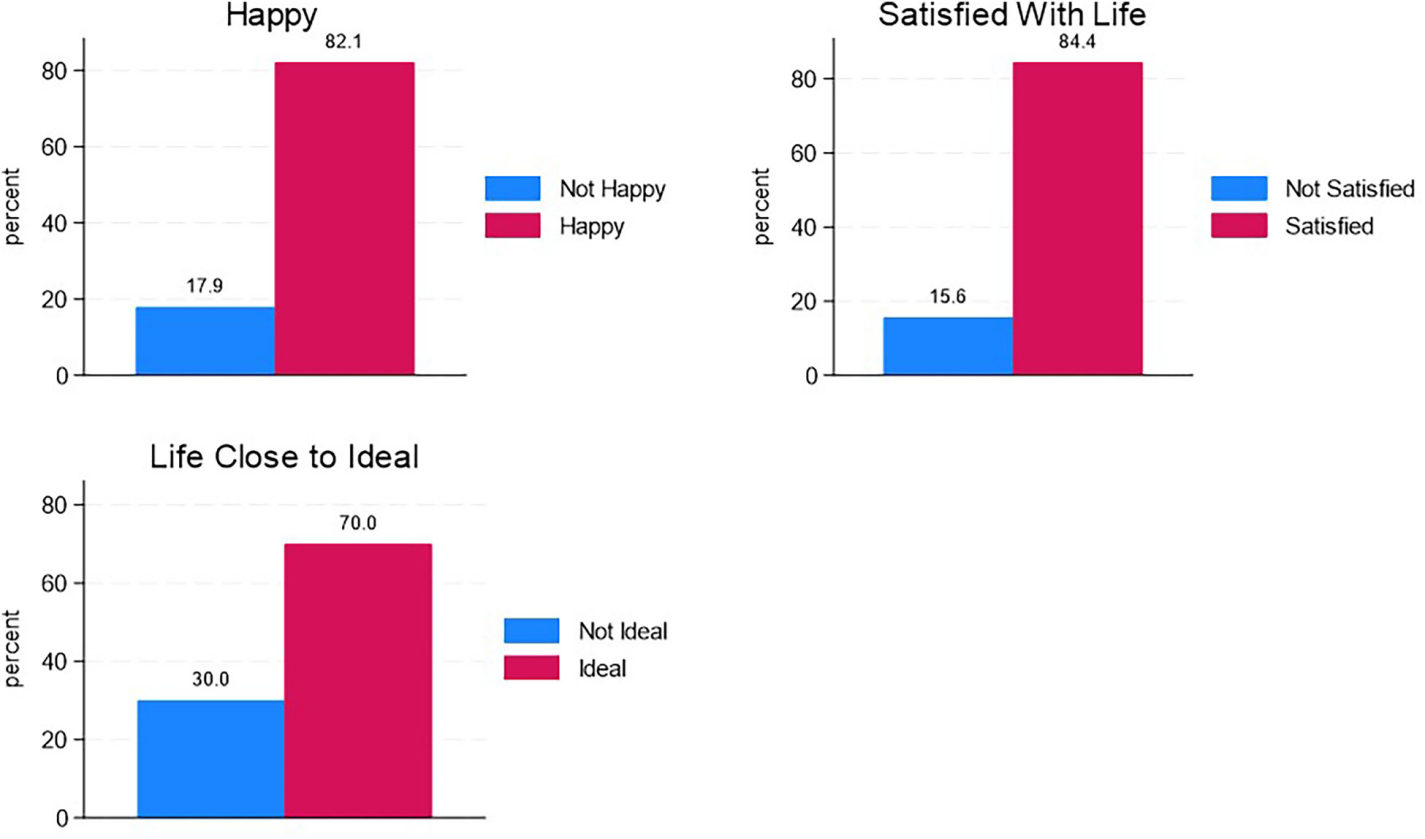
Distribution of dependent variables.

Summary statistics of variables are presented in Figure 2 and in Table 2. In Figure 2, it is observed that 82.02% of clergy members reported feeling happy, and 84.39% expressed satisfaction with their lives. Approximately 70% indicated that their life was close to the ideal. Table 2 shows that the majority of clergy members were male (84.30%), of white ethnicity (74.10%), classified as overweight or obese (79.90%), possessed formal pastoral training (61.75%), and identified as white conservative, evangelical, or fundamentalist (49.32%). Furthermore, 47.35% of clergy members reported a middle-income status. Only 16.21% of the data was collected after the onset of the COVID-19 pandemic. Approximately 37.08% of clergy members contemplated leaving their religious work, 35.37% experienced doubts about their faith, ranging from occasional to constant, and 25.06% reported feeling inadequately supported by their congregation.

**Table 2.** Summary Statistics of Variables.

Variables	Weighted percentage
Sex	
Male	84.30
Female	15.70
BMI	
Normal BMI	20.10
Overweight/Obese	79.90
Education on Mental Illness	
Not at all	28.15
Once or more	71.85
Education	
No College or formal pastoral or ministerial training	4.63
All degrees except MDiv	33.62
Formal Pastoral or ministerial training/MDiv	61.75
Race	
White or Am Ind	74.10
Black	21.10
Hispanic	3.49
Asian/PI	1.32
COVID	
No	83.79
Yes	16.21
Household Income	
Low	28.90
Middle	47.35
High	23.74
Contemplated Leaving religious work	
Never	62.92
Leave	37.08
Doubts about faith	
Never or do not have a religious faith	64.63
Sometimes to all the time	35.37
Feeling cared for	
Low	25.06
High	74.94
Religious Tradition	
Roman Catholics	3.71
White conservative, evangelical, or fundamentalist	49.32
Black Protestant	17.82
White liberal or moderate	22.66
Non-Christian	6.50

*Note.* Weighted values.

**Figure 3.**
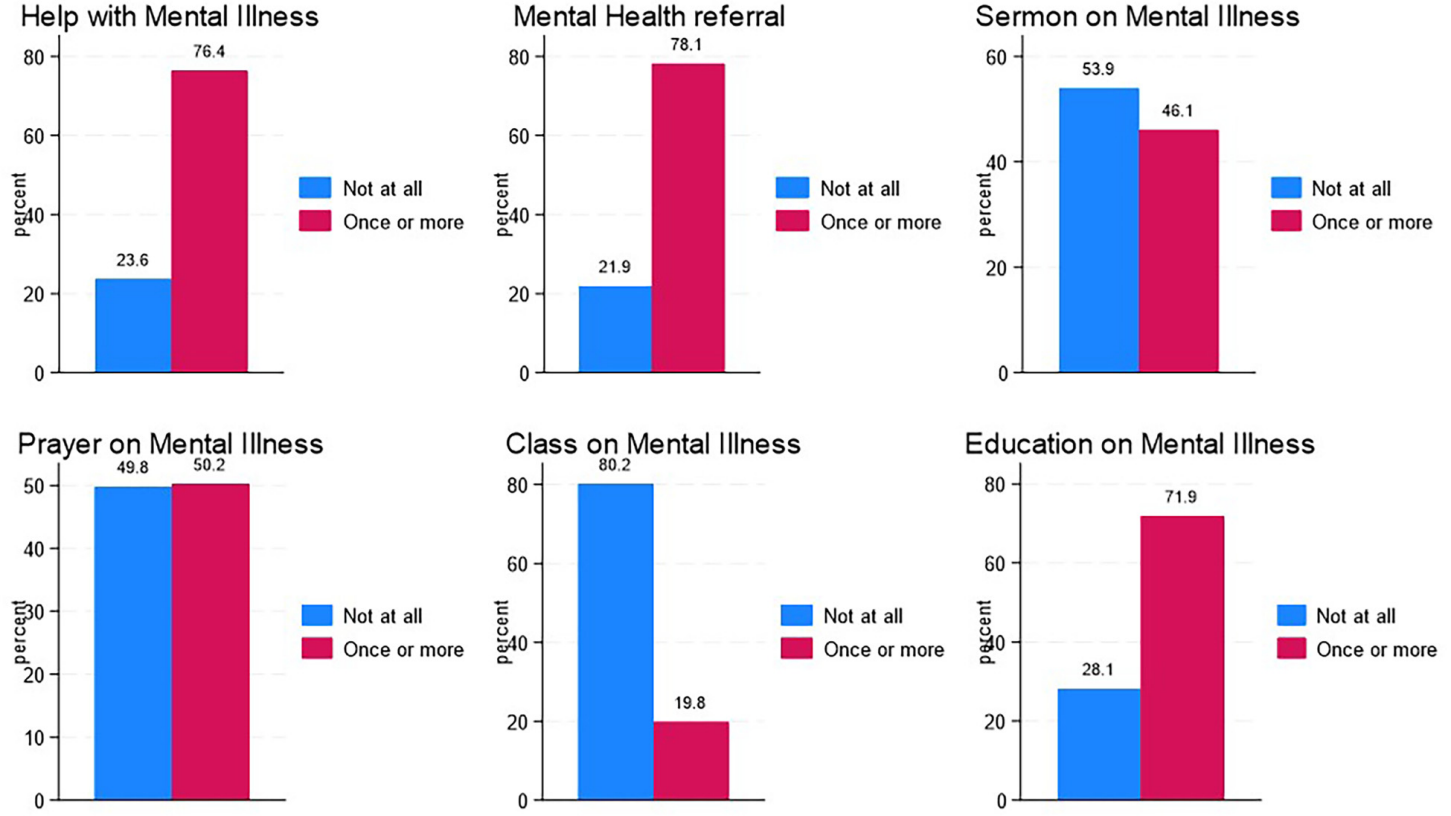
Distribution of mental health activities.

As shown in Figure 3, among participating clergy members, 76.36% were approached by congregants for assistance with mental illness on at least one occasion. In contrast, 78.15% of clergy members encouraged someone at least once to seek help from a mental health professional in the past year. In the same time period, 71.85% of clergy members engaged in mental health-related activities, such as reading books, attending classes, or seeking resources on mental illness. Additionally, 46.06% preached a sermon on mental illness, 50.23% led prayers or healing focused on mental health, and 19.83% organized classes on mental illness for their congregants.

### *Multivariate Regression Results*

#### Model 1: Happiness Among Clergy Members

In Model 1 of Table 3, clergy who encouraged congregants to seek help from a mental health professional decreased the odds of happiness by a factor of 0.10 [95% CI: 0.02, 0.56]. Clergy who considered leaving their work had reduced odds of happiness by a factor of 0.29 [95% CI: 0.13, 0.64], compared with those who did not contemplate leaving their work. Similarly, clergy who had doubts about their faith had decreased odds of happiness by a factor of 0.38 [95% CI: 0.16, 0.91] compared with those without doubts about their faith. Clergy members who reported receiving high levels of care from their congregants had significantly higher odds of happiness, with an odds ratio of 8.59 [95% CI: 3.71, 19.90], compared with those who reported receiving low levels of care. Black clergy had decreased odds of happiness compared with Whites or American Indian clergy by a factor of 0.02 [95% CI: 0.00, 0.16].

**Table 3.** Multivariate Regression of Clergy Happiness, Satisfaction, and Life Close to Ideal Controlling for Covariates.

	(Model 1)	(Model 2)	(Model 3)
Variables	Happy	Satisfied with life	Life close to ideal
Help with Mental Illness: *[Ref: Not at all]*			
Once or more	2.08	0.66	1.19
	(0.61–7.12)	(0.24–1.83)	(0.63–2.24)
Mental Health referral: *[Ref: Not at all]*			
Once or more	0.10***	1.09	1.71
	(0.02–0.56)	(0.38–3.18)	(0.89–3.32)
Sermon on Mental Illness: *[Ref: Not at all]*			
Once or more	1.74	1.46	0.51***
	(0.81–3.75)	(0.73–2.92)	(0.31–0.83)
Prayer on Mental Illness: *[Ref: Not at all]*			
Once or more	1.26	2.07**	1.02
	(0.64–2.47)	(1.13–3.80)	(0.66–1.59)
Class on Mental Illness: *[Ref: Not at all]*			
Once or more	0.74	0.37**	1.20
	(0.28–1.92)	(0.17–0.82)	(0.65–2.20)
Education on Mental Illness: *[Ref: Not at all]*			
Once or more	0.98	0.52*	0.79
	(0.42–2.28)	(0.23–1.13)	(0.46–1.37)
Gender: Ref *[Male]*			
Female	4.55***	1.44	0.42***
	(1.58–13.08)	(0.69–3.02)	(0.23–0.75)
BMI: *[Ref: Normal weight]*			
Overweight/Obese	0.50	0.65	0.46***
	(0.20–1.26)	(0.31–1.34)	(0.25–0.82)
Education: *[Ref: No College or formal pastoral or ministerial training]*			
All degrees except MDiv	8.63**	6.48***	4.28***
	(1.16–64.06)	(1.72–24.43)	(1.56–11.77)
Formal Pastoral or ministerial training/MDiv	16.70***	13.47***	3.87***
	(1.96–142.03)	(3.60–50.34)	(1.46–10.24)
Race: *[Ref: White or Am Ind]*			
Black	0.02***	7.47*	5.93**
	(0.00–0.16)	(0.87–64.13)	(1.27–27.64)
Hispanic	4.95	2.05	1.55
	(0.51–48.11)	(0.39–10.87)	(0.53–4.53)
Asian/PI	0.20	0.55	2.20
	(0.01–4.92)	(0.06–5.54)	(0.33–14.59)
COVID: *[Ref: Yes]*			
No	4.86***	1.74	0.80
	(1.64–14.44)	(0.74–4.11)	(0.44–1.45)
Income: *[Ref: Low Income]*			
Middle	1.41	1.59	3.48***
	(0.50–3.93)	(0.73–3.45)	(1.99–6.08)
High	0.65	1.17	2.02**
	(0.23–1.89)	(0.52–2.63)	(1.10–3.69)
Contemplated Leaving religious work: *[Ref: Never]*			
Once in a while to often	0.29***	0.21***	0.28***
	(0.13–0.64)	(0.11–0.40)	(0.17–0.44)
Doubts about faith: *[Ref: Don’t have a religious faith]*			
Sometimes to all the time	0.38**	0.22***	0.42***
	(0.16–0.91)	(0.11–0.45)	(0.26–0.70)
Feeling cared for: *[Ref: Low]*			
High	8.59***	4.47***	3.48***
	(3.71–19.90)	(2.43–8.22)	(2.16–5.61)
Religious Tradition*: [Ref: Roman Catholics]*			
White conservative, evangelical, or fundamentalist	0.34	0.41	0.71
	(0.08–1.39)	(0.03–5.25)	(0.22–2.32)
Black Protestant	32.93***	0.06*	0.22
	(3.15–344.00)	(0.00–1.49)	(0.03–1.47)
White liberal or moderate	0.11***	0.18	1.02
	(0.03–0.43)	(0.01–2.34)	(0.30–3.49)
Non-Christian	0.14	0.15	2.36
	(0.01–1.48)	(0.01–2.36)	(0.54–10.38)
Constant	5.26	6.13	0.64
	(0.34–82.01)	(0.34–109.04)	(0.13–3.22)
Observations	636	636	636

*p < 0.05, **p < 0.01, ***p < 0.001.

Female clergy exhibited significantly increased odds of happiness compared with their male counterparts by a factor of 4.55 [95% CI: 1.58, 13.08]. Clergy with degrees other than an MDiv with some form of formal pastoral, ministerial training or an MDiv compared with those without any college or formal training showed significantly increased odds of happiness by a factor of 8.63 [95% CI: 1.16, 64.06] and 16.70 [95% CI: 1.96, 142.03] respectively. Black Protestants had increased odds of being happy. In contrast white liberal or moderate clergy had a decreased odds of being happy by a factor of 32.93 [95% CI: 3.15, 344.00] and 0.11 [95% CI: 0.03, 0.43], respectively, compared with White conservative, evangelical, or fundamentalist clergy.

#### Model 2: Life Satisfaction Among Clergy

In model 2 of Table 3, clergy who organized a class on mental illness had decreased odds of being satisfied with life by a factor of 0.37 [95% CI: 0.17, 0.82] compared with those who did not organize a class focused on mental illness once or more in the past 12 months. Clergy who led a prayer session focused on mental illness had higher odds of being satisfied with life by a factor of 2.07 [95% CI: 1.13, 3.80] compared with those who did not lead a prayer session focused on mental illness. Further, clergy members who considered leaving their religious work had lower odds of being satisfied with life by a factor of 0.21 [95% CI: 0.11, 0.40] compared with clergy who did not consider leaving their religious work. Clergy who had doubts compared with those who had no doubts about their faith had lower odds of being satisfied with life by a factor of 0.22 [95% CI: 0.11, 0.45]. Higher odds of being satisfied with life by a factor of 4.47 [95% CI: 2.43, 8.82] was found among clergy who reported receiving high levels of care from their congregants compared with clergy who reported receiving low levels of care from their congregants. Clergy with degrees other than MDiv and those with formal pastoral or ministerial training or MDiv compared with those with no college, formal pastoral or ministerial training, had higher odds of being satisfied with life by a factor of 6.48 [95% CI: 1.72, 24.43] and 13.47 [95% CI: 3.60, 50.34], respectively.

#### Model 3: Life Close to Ideal

In model 3 of Table 3, clergy who preached a sermon on mental illness in the past 12 months had lower odds of having life close to the ideal by a factor of 0.51 [95% CI: 0.31, 0.83] compared with clergy who did not give a sermon on mental illness at least once in the past 12 months. Clergy who considered leaving religious work compared with those who never considered it had significantly lower odds of their life being close to ideal by a factor of 0.28 [95% CI: 0.17, 0.44]. Clergy who had doubts about their faith had decreased odds of their life being close to ideal by a factor of 0.42 [95% CI: 0.26, 0.70] in comparison with those who never doubted their faith or did not have a religious faith.

On the other hand, clergy who reported receiving high levels of care from their congregants compared with those who reported receiving low levels of care from their congregants had substantially higher odds of their life being close to ideal by a factor of 3.48 [95% CI: 2.16, 5.61]. Black clergy had higher odds of their life being close to ideal than Whites or American Indian clergy by a factor of 5.93 [95% CI: 1.27, 27.64]. In contrast to male clergy, female clergy exhibited decreased odds of their life being close to the ideal by a factor of 0.42 [95% CI: 0.23, 0.75]. Clergy members with educational backgrounds other than an MDiv degree and those with formal pastoral or ministerial training demonstrated higher odds of their lives being close to the ideal by a factor of 4.28 [95% CI: 1.56, 11.77] and 3.87 [95% CI: 1.46, 10.24], respectively, in comparison with clergy with no college education or formal pastoral/ministerial training. Overweight or obese clergy had decreased odds of their life being close to ideal by a factor of 0.46 [95% CI: 0.25, 0.82] compared with clergy with normal body weight. Clergy in the middle- and higher-income brackets demonstrated a significantly greater probability of living a life close to the ideal when contrasted with clergy in the lower-income category. The odds ratios were 3.48 [95% CI: 1.99, 6.08] and 2.02 [95% CI: 1.10, 3.69], respectively.

## Discussion

Our findings indicate that clergy engaged in various mental health activities in response to the mental health needs of their congregants. These activities included encouraging congregants to seek mental health professionals, reading books on mental illness, leading prayer services focused on mental health, preaching sermons on the topic, and seeking external educational resources.

Specifically, clergy who referred congregants to mental health professionals reported decreased happiness. In addition, clergy who provided prayer or healing for mental health illness had higher life satisfaction, and clergy who preached a sermon on mental illness reported life being less close to ideal. We also found that clergy who reported receiving high levels of care from their congregants reported greater happiness, life satisfaction, and life closer to the ideal. Clergywomen were likelier to have increased happiness but life less close to the ideal. In contrast, clergy with formal pastoral training had increased happiness, life satisfaction, and life close to the ideal. Clergy experiencing greater doubts or thinking about leaving work had lower happiness, satisfaction, and less-than-ideal life.

These findings are consistent with the Job Demands-Resources Model, which suggests that when congregants turn to clergy for assistance with mental health concerns, the specific actions and activities undertaken by clergy in response to this demand can significantly impact various aspects of their well-being. In this context, the model suggests that clergy's responses to congregants’ mental health needs may play a crucial role in shaping clergy levels of life satisfaction, overall happiness, and the extent to which they perceive their lives as being close to their personal ideals. Specifically, congregants seeking help with mental health issues represent a specific demand or challenge within the professional role of clergy. The activities clergy engage in, in turn, influence well-being outcomes, including their satisfaction with life, overall happiness, and their sense of living a life that aligns with their ideal values and aspirations.

These results are essential for furthering our understanding of how the mental health demands of congregants may influence clergy happiness, life satisfaction, and their perception of life close to the ideal, as well as the mental health activities utilized by clergy to support their congregants. As trusted leaders in various communities, clergy often serve as a primary source of support for congregants facing emotional or mental challenges (Hays, 2018). For example, Wang and Colleagues (2003) found that 25% of U.S. adults with mental health issues including substance abuse seek mental health support from their religious leaders (Wang et al., 2003; Wong et al., 2018). Among historically underrepresented communities, congregants are more likely to turn to faith leaders for assistance when burdened with mental health challenges (Bohnert et al., 2010), as shown in a study where around 40% of African Americans consider clergy their primary source of help for dealing with depression (Anthony et al., 2015).

Our discovery that female clergy exhibited higher levels of happiness and perceived life as not close to the ideal compared with male clergy aligns with previous research (Holleman, 2023). It may well be that clergywomen do not view their life as ideal yet are more likely to be happy due to their ability to manage job-related stress effectively. In one study, female clergy reported greater life satisfaction than their male counterparts, suggesting that socially conditioned gender-specific job values may account for this gender paradox (McDuff, 2001). Clergywomen may derive satisfaction from aspects of their work or personal lives that differ from the traditional expectations or values associated with their role. However, it's worth noting that this study did not identify a statistically significant association between gender and life satisfaction, highlighting the complexity of the factors influencing clergy members’ well-being and life perceptions.

As respected community leaders, clergy's position on mental health can influence congregants’ views of mental health and their actions to seek support (Campbell, 2021). Conveying denominational and institutional opinions about mental health, clergy are a pillar of their religious organization (Ward et al., 2013). Therefore, clergy must be trained in identifying, supporting, and referring congregants experiencing mental health illness to mental health professionals. As present research is limited, our study findings elucidate the activities clergy engage in when addressing the mental health needs of congregants.

Our results are similar to other studies reporting that church leaders refer congregants to mental health services and providers (Allen et al., 2010; Heseltine-Carp & Hoskins, 2020; Wong et al., 2018). However, barriers to providing treatment referrals in religious organizations exist, including stigma and insufficient staff or resources (Jang et al., 2014; Mathison, 2016; Wong et al., 2018). Clergy may be more effective in referring congregants with higher risk concerns such as suicidal ideation, substance abuse, or psychosis than depression and anxiety (Heseltine-Carp & Hoskins, 2020).

Our research outcomes concerning clergy members who reported feeling highly cared for align with a broader body of research. Specifically, we observed that clergy who reported receiving high levels of care from congregants exhibited higher levels of happiness, greater life satisfaction, and a stronger sense that their lives were close to their ideal. In a study by Edwards et al. (2021), it was found that clergy who perceived themselves as receiving greater levels of social support were less likely to experience symptoms of depression. This correlation underscores the significant role that social support plays in the well-being of clergy members. Feeling cared for and supported by their congregants and communities appears to contribute positively to various dimensions of clergy members’ well-being, including emotional well-being, life satisfaction, and the perception of leading a life in accordance with their ideals. This finding highlights the reciprocal nature of support within religious communities, where both clergy and congregants benefit from strong, supportive relationships.

Clergy members might experience feelings of inadequacy when they must refer congregants with mental health challenges to external mental health providers outside the church, as they may perceive their abilities as insufficient to meet those needs entirely. This can in turn negatively influence their happiness as shown in our findings and other studies (Bledsoe et al., 2011). Interestingly, our findings also indicate that clergy members who conducted prayer or healing services specifically focused on mental illness tended to report higher levels of life satisfaction. The intriguing aspect of these two findings is the potential disconnect between them, which hints at the intricate interplay between clergy members feeling both equipped and unequipped when addressing the mental health needs of their congregants. In other words, this suggests that clergy members navigate a complex landscape where they may sometimes feel competent and effective in providing support for members’ mental health issues through activities like prayer services, while at other times, they grapple with feelings of inadequacy when the complexity of certain cases necessitates external professional help. It highlights the importance of recognizing the various ways in which clergy members engage with and respond to congregants’ mental health challenges, acknowledging that both their feelings of capability and limitations can coexist (Swain, 2016) within their pastoral care efforts.

Clergy can leverage their trusted position in local communities to partner with mental health professionals and build a therapeutic alliance. By providing mental health resources such as names and contact information for mental health professionals, support groups, and treatment facilities, clergy can build this alliance with extra support to meet the needs of congregants experiencing anxiety, depression, substance abuse, and psychosis. Responses to congregants’ mental health struggles should be met with cultural and spiritual sensitivity by clergy (Lloyd & Waller, 2020). Mental health training could increase clergy competence in addressing the emotional needs of congregants, and they should be careful not to perpetuate stigma (Lehmann et al., 2022).

### *Limitations*

The sample predominantly consisted of white and male participants, limiting the generalizability of the findings. Moreover, the data collection spanned pre- and post-COVID-19 periods, potentially influencing the responses of clergy members. It is important to interpret this study cautiously since it is a cross-sectional design, which does not establish causality, and the observed relationships could be influenced by unaccounted variables in our analysis.

## Conclusions

Clergy use various mental health activities to support congregants with mental health needs, including prayer, preaching on mental health, referral to mental health professionals, and reading mental health-related materials. Formal pastoral training is associated with greater happiness, life satisfaction, and a sense of life closer to the ideal, emphasizing the importance of comprehensive pastoral training to equip clergy to identify, support, and refer congregants experiencing mental health disorders to mental health professionals. Further investigation through a comparative study to examine the variations in available mental health resources, life satisfaction, and happiness among racially diverse clergy groups is necessary to provide culturally sensitive mental health resources and training for clergy members. Specifically, more clergy from historically underrepresented populations who experience more significant mental health disparities due to persistent social and health inequities should be considered in future research.
